# Develop an empirical flow rate correlation to model wellbore storage phenomenon for wells produced at a constant wellhead pressure

**DOI:** 10.1038/s41598-023-44678-3

**Published:** 2023-10-18

**Authors:** Mobarakeh Mohammadpour, Parisa Behnoud, Mohammad Reza Khorsand Movaghar

**Affiliations:** Department of Petroleum Engineering, Amirkabir University of Technology (Tehran Polytechnic), 424 Hafez Avenue, Tehran, P.O. Box 15875-4413, 1591634311 Iran

**Keywords:** Fossil fuels, Engineering

## Abstract

Well-test operation with a constant flow rate for long-time production is not typical in practice. Therefore, reservoirs frequently produce fluids with constant pressure at the wellhead, into a constant pressure separator or pipeline. During the production under this condition, the pressure drop at the wellbore varies as a function of the rate because of changes in the friction flow; therefore, the wellbore formation pressure is not constant. As a consequence, the wellbore storage effect emerges and should be incorporated in the modeling of fluid flow under production at a constant wellhead pressure. All available methods considering the wellbore storage effect can merely be applied for production under the constant rate condition. Moreover, well-test modeling under these circumstances needs a specific function for production rate, which made this type of well-testing less common. Even though there are exact solutions for production under the constant wellbore pressure in the Laplace domain, their inverse transform is quite complex and cannot be applied for modeling the wellbore storage effect. In this study, a new function is introduced as an approximate solution of the diffusivity equation, for a well that produces at a constant wellbore pressure, using multiple steps of the optimization process. Consequently, an efficient correlation is developed for flow rate about or concerning this condition. Next, the effect of the wellbore storage at constant pressure well-test operation was enumerated for the first time, which was commonly neglected in previous studies. This endeavor concentrated on modeling the two types of well-test operation, producing at constant wellhead pressure and, pressure build-up following the production at a constant wellhead pressure (constant pressure Build-up test). The results were also verified with numerical simulation as well as field data further confirming the excellent accuracy of developed solutions.

The development of models in an attempt to precisely predict fluid flow in underground reservoirs is of great importance. Regarding the significance of the governing equations in future production planning and pressure behavior study, including properties such as well bore storage, porosity, permeability, skin factor, etc., is necessary to practicably estimate production at constant pressure or constant rate.

The well testing methods are commonly devised for production at a constant rate. However, considering the difficulties in maintaining constant flow rate especially for a long time, most of the wells produce at a constant wellhead pressure^1^. Such cases could be considered for wells in reservoirs with low permeability like unconventional reservoirs, geothermal reservoirs under fluid drive with a back-pressured turbine, open wells including artesian water wells which often produce at a constant pressure^2^.

With science thriving on analogy, while the methods used for the interpretation of pressure build-up and pressure draw-down tests are not suitable for wells at constant wellhead pressure, these interpretations can be used through comparison^3^. Considering the challenges in the modeling of the well-test operation under constant wellhead pressure and wellbore storage condition, including the necessity of a specific function for production rate, this type of well-testing has received less attention.

This study aims at providing a new method for modeling the wellbore storage effect for well-testing at a constant wellhead pressure. To this end, firstly, the previous theories and studies on modeling well-testing at constant wellbore pressure will be discussed.

Van Everdingen and Hurst^4^ used Laplace transformation as a new tool for solving problems encountered in the study of flowing fluids. They demonstrated that the constant terminal rate and constant terminal pressure modes are interrelated, where with knowing the operational condition of one mode, the other can be determined. They used the Laplace transform method, and consequently the *Mellin’s inversion* formula to find final form of flow rate under production at a constant wellbore pressure as presented in Table 1.

**Table 1:** Concise information on significance, developed methods and/or final correlation for flow rate for a well producing under constant-wellbore pressure.

Reference	Significance/method	Correlation for flow rate
Van Everdingen and Hurst^4^	· Exact analytical solutions · Using Laplace method and *Mellin’s* inversion formula	${\text{q}}_{{\text{D}}} \left( {{\text{t}}_{{\text{D}}} } \right) = \frac{4}{{\pi^{2} }}\int_{0}^{\infty } {\frac{{\left( {e^{{ - u^{2} t_{D} }} } \right)du}}{{u \left[ {J_{0}^{2} \left( u \right) + Y_{0}^{2} \left( u \right)} \right]}}}$
Jacob and Lohman^5^	· Analytical solution · Using the Green’s function and heat transfer analogy	${\text{ q}}_{{\text{D}}} \left( {{\text{t}}_{{\text{D}}} } \right) = \frac{{4t_{D} }}{\pi }\int_{0}^{\infty } {\chi e^{{ - t_{D} \chi^{2} }} \left( {\frac{\pi }{2} + tan^{ - 1} \left[ {\frac{{Y_{0} \left( x \right)}}{{J_{0} \left( x \right)}}} \right]} \right)}$
Clegg^6^	· Approximation solution · Using inversion formula of the *Schapery method* · For large values of time	${\text{ q}}_{{\text{D}}} \left( {{\text{t}}_{{\text{D}}} } \right) \approx \sqrt {\frac{1}{{2t_{D} }} \times } \frac{{K_{1} \left( {\sqrt {\frac{1}{{2t_{D} }}} } \right)}}{{K_{0} \left( {\sqrt {\frac{1}{{2t_{D} }}} } \right)}}$
Kleppe and Cekirge^3^	· Approximation solution · Using Laplace transform and superposition principle · For large values of time	$$\begin{aligned} & {\text{for}}\quad { }10^{2} \le {\text{t}}_{{\text{D}}} \le 10^{6} : \\ & {\text{q}}_{{\text{D}}} \left( {{\text{t}}_{{\text{D}}} } \right) = 0.0500 + 0.313\left( {\frac{1}{{{\text{t}}_{{\text{D}}} }}} \right)^{0.2043} { } \\ & {\text{for}}\quad 4.10^{4} \le {\text{t}}_{{\text{D}}} \le 10^{12} : \\ & {\text{q}}_{{\text{D}}} \left( {{\text{t}}_{{\text{D}}} } \right) = 0.0250 + 0.183\left( {\frac{1}{{{\text{t}}_{{\text{D}}} }}} \right)^{0.1040} \\ \end{aligned}$$
Uraiet and Rajagopal^7^	· Numerical solutions · Using Finite Difference method	–
Ehlig Economides and Ramey^8^	(a) Semi-Analytical solutions · Using Laplace transform and Stehfest as a numerical inversion algorithm (b) Approximation solution for large values of time	(a) – (b) Approximation Formula: $$\begin{aligned} & {\text{for }}\quad 8 \times 10^{4} \le {\text{t}}_{{\text{D}}} \& Error < 1\% \\ & {\text{q}}_{{\text{D}}} \left( {{\text{t}}_{{\text{D}}} } \right) = \left[ {\frac{1}{2}\left( {\ln {\text{t}}_{{\text{D}}} + 0.80907 + 2{\text{s}}} \right)} \right]^{ - 1} \\ \end{aligned}$$
Lu et al.^9^ and^10^	Single well^9^ and Multiple well^10^ · Semi-Analytical solutions · Combinations of Dirac delta function, Bessel functions, Laplace transform, Green’s function · Stehfest as a numerical inversion method for Laplace transform	–
Vivas-Crus et al.^11^	Modeling of finite fracture reservoirs Using complex mathematical model: Joint Laplace- Hankel Transform	Validation: Check with the inverse formula of flow rate proposed by Van Everdingen and Hurst^4^
Zhiqiang Fan and Rishi Parashar^11^	Pumping test for confined aquifer Including Poroelastic effect and wellbore storage Using Laplace transform and Stehfest as a numerical inversion algorithm	–
In this study	Wellbore storage effect Approximate solution using the Combination of Variable method and Optimization process	$$\begin{aligned} & {\text{q}}_{{\text{D}}} \left( {{\text{t}}_{{\text{D}}} } \right) = - \left( {{\text{b}}\left( {{\text{t}}_{{\text{D}}} } \right) + \frac{{2{\text{e}}^{{ - \frac{1}{{4{\text{ t}}_{{\text{D}}} }}}} }}{{{\text{Ei}}\left[ { - \frac{1}{{4{\text{t}}_{{\text{D}}} }}} \right]}}} \right) \\ & b\left( {{\text{t}}_{{\text{D}}} } \right) = 0.001 + \frac{0.420}{{{\text{t}}_{{\text{D}}}^{0.605} }} \\ \end{aligned}$$

According to the above discussion, the achieved correlation for flow rate is very complex and with no simple approximation available, it must be solved numerically. Therefore, the main difficulty is that the solutions of pressure and flux (into the well) equations in Laplace space cannot be easily inverted to analytical functions in real space. Consequently, only numerically tabulated solutions are available. Moreover, simplifications over time domain for small- and large-time intervals applied in the literature, restricts the solution to the specified conditions.

Using the Green’s function and heat transfer analogy, Jacob and Lohman^5^ obtained a solution for the production rate at a constant wellbore pressure as presented in Table 1. With application of a complicated numerical method the production rate using the proposed correlation was successfully estimated.

Later, Clegg^6^ implemented an approximation technique in an attempt of finding the Laplace inverse for pressure equation. Clegg implemented the *Schapery method* as an approximate solution for large values of time and found the flow rate correlation as presented in Table 1.

It should be noted that the suggested approximate solution is applicable as long as the *Schapery method* large time assumption is valid.

Kleppe and Cekirge^3^ developed an approximate solution to the constant pressure test using both Laplace transformation and the superposition principle. After some simplifications, they rendered the flow rate correlation for two periods of time as represented in Table 1. Therefore, the method is only applicable for large times. In addition, the flux integral was calculated approximately using specific functions.

Uraiet and Rajagopal^7^ introduced a simple procedure for analyzing pressure build up after constant pressure production using finite difference method.

Ehlig-Economides and Ramey^8^ derived well-testing equations at a constant wellbore pressure for a reservoir with an infinite outer boundary, closed outer boundary, and outer boundary at a constant pressure using the *Laplace transforms* and *Stehfest numerical* algorithm.

Economides^8^ proposed an approximate function for production rate at a constant wellbore pressure and calculated the pressure build-up using the superposition principle. The proposed rate function for the infinite reservoir provided in equation is applicable with an error rate of less than 1% at ${t}_{D}>8\times {10}^{4}$ as presented in Table 1.

Recently, Lu et al.^9^ proposed a new mathematical model for production performance of a well producing at constant bottom hole pressure using Dirac delta function, Bessel functions and Laplace transform; however, Stehfest was used as a numerical inversion method with the results mostly provided for decline curves analysis. Subsequently, Lu et al.^10^ applied this mathematical model to predict the production performance of multiple-well system during boundary-dominated flow period in a closed rectangular reservoir.

Moreover, Vivas-Crus et al.^11^ have investigated the modeling of finite fracture reservoirs with various inner and outer boundary conditions in which production at a constant bottom hole pressure was also included. They used a complex mathematical model named Joint Laplace- Hankel Transform to solve the equations and finally, for validation they checked the results with the inversion of Laplace transform formulas (which had been previously introduced by Van Everdingen and Hurst^4^).

Well test analysis and interpretation for confined aquifers/reservoirs always face with own challenges. Recently a new analytical solution was introduced for pumping test in low-permeability aquifers that simultaneously accounts for the poroelastic effect and wellbore storage; The authors used the Laplace transform to solve the governing equation in plane strain poroelasticity. The outcomes show that wellbore storage significantly delays drawdowns at the pumping well and tends to mask the poroelastic effect in the vicinity of the pumping well. As a result, the pumping-induced poroelastic deformation of an aquifer would impact the interpretation result, especially at early times. Hence in the hard rock aquifer/reservoirs, the poroelasticity effect is vital to include not only in a geomechanical model (which reveals the mechanical behavior of rock and wellbore e.g. in modeling wellbore stability) but also in modeling draw-down tests^12,13^.

Based on previous studies, although Laplace transforms^4^ and Green’s function^5^ have been used frequently leading to exact solutions however their inverse transforms can be quite complicated resulting in complex numerical integrals or numerical integral tables as final solutions.

As a result, with no specific function being obtained for the constant production rate well-testing, demonstrates that these types of solutions cannot be applied for modeling the wellbore storage effect.

Although Economides developed a particular rate function, the proposed function was applicable for long periods and was not applicable for calculations during the wellbore storage time interval (i.e., early time production).

Hence, In the second step, an infinite acting radial reservoir under constant pressure well-testing operation is considered and modeled. While the method of the *combination of variables* cannot be used to solve the diffusivity equation, it is applied to provide an initial format for an approximate solution. Therefore, based on the initial format introduced by *combination of variables*, and discrete data from Van Everdingen and Hurst^4^ multiple steps of optimization processes were implemented. This approach leads to propose an empirical correlation to the pressure distribution as a function of time and radius, and ultimately the transient rate decline function.

In third step, incorporating the proposed function, the effect of wellbore storage is mathematically modeled, leading to new equations for the two types of well-test operation under consideration. One for production under constant wellhead pressure, and another for pressure build-up following a constant wellhead pressure production which was commonly neglected in the previous studies. It should be noted that given the wellbore storage effect at early times, a certain efficient rate function is required to be used at early times.

Finally, for model validation, the results of the model, in each stage, are compared with those obtained from well-testing numerical simulation.

## Analysis

A well, producing at constant pressure, exhibits a transient rate decline which can be analyzed using methods analogous to those for constant-rate flow. By this assumption that formation is not transverse anisotropic (radial and vertical hydraulic conductivities are not equal) and in this regard the vertical hydraulic conductivity could be ignored versus radial hydraulic conductivity, the pressure behavior in a radial reservoir can be described by the diffusivity equation as follows:

1 $$\frac{{\partial }^{2}P}{\partial {r}^{2}}+\frac{1}{r}\frac{\partial P}{\partial r}=\left(\frac{\phi \mu c}{k}\right)\frac{\partial P}{\partial t}$$

where $\mathrm{P}$ is the pressure, $\mathrm{r}$ is the distance from the well, $\mathrm{t}$ is the time, $\mathrm{k}$ is the permeability, $\upphi$ is the porosity, $\upmu$ is the viscosity and $\mathrm{c}$ is the total compressibility.

The above equation can be simplified using Eqs. (2)–(6) in dimensionless formulation:

2 $${P}_{D}=\frac{{P}_{i}-P\left({r},t\right)}{{P}_{i}-{P}_{w}}$$

3 $${r}_{D}=\frac{r}{{r}_{w}}$$

4 $${r}_{De}=\frac{{r}_{e}}{{r}_{w} }$$

5 $${t}_{D}=\frac{k}{\phi \mu c{ r}_{w}^{2}}t$$

6 $$\frac{1}{{r_{D} }}\frac{\partial }{{\partial r_{D} }}\left( {r_{D} \frac{{\partial P_{D} }}{{\partial r_{D} }}} \right) = \frac{{\partial P_{D} }}{{\partial t_{D} }}$$

where ${P}_{D}$ is the dimensionless pressure, ${P}_{i}$ is the initial reservoir pressure, ${P}_{w}$ is the wellbore pressure, ${r}_{D}$ is the dimensionless radius, ${r}_{De}$ is the dimensionless reservoir radius, ${r}_{e}$ is reservoir radius, ${r}_{w}$ is the wellbore radius and ${t}_{D}$ is the dimensionless time.

The initial and boundary conditions are listed in Eqs. (7), (8) and (9):

7 $${\varvec{I}}.{\varvec{C}}. {\left.P\right|}_{t=0}={P}_{i} \to { \left.{P}_{D}\right|}_{{t}_{D}=0}=0$$

8 $${\varvec{B}}.{\varvec{C}}.\#1\boldsymbol{ }\boldsymbol{ }\boldsymbol{ }\boldsymbol{ } P={P}_{w} \to {\left.{P}_{D}\right|}_{{r}_{D}=1}=1$$

9 $${\varvec{B}}.{\varvec{C}}.\#2 {\left.P\right|}_{{r}_{e}\to \infty }={P}_{i} \to {\left.{P}_{D}\right|}_{{r}_{De}\to \infty }=0$$

It should be noted that, since the well-test operation is always performed for a limited and finite period, the corresponding time is not necessarily long enough to let the leading edge of the pressure pulse to reach to the outer boundary of the reservoir. Thus, for modelling the well-test process, the external radius of reservoir is considered large enough (infinite radius) in such a way that the pressure is supposed to be equal to initial reservoir pressure P_i_ at outer boundary condition, as represented in B.C. #2.

The problems with reservoirs with infinite radius (infinite-acting reservoir) are typically solved with the help of *Laplace transforms* and *Green’s functions*. Van Everdingen and Hurst^4^ used the Laplace transform while Jacob and Lohman^5^ employed the Green’s function for well-test modeling at a constant wellbore pressure. As discussed in the introduction part they used numerical methods to calculate the inverse Laplace transform and the rate production integral, respectively.

Due to their complexity, the inverse Laplace transforms and integral functions cannot be used in mathematical calculations, specifically in obtaining pressure equations during the production at a constant wellhead pressure as well as in the pressure build-up test following the production at a constant wellhead pressure (when the well is shut-in after production at a constant wellhead pressure, and conditions are turned into zero rate production). Therefore, this study aims at developing a specific production rate function.

Referring to the diffusivity equation (Eq. 6), the general solution to the diffusivity equation can be obtained through the combination of variables method^14^, as follows:

10 $$P_{D} = C_{1} \cdot Ei\left( { - \frac{{r_{D}^{2} }}{{4t_{D} }}} \right) + C_{2}$$

where C_1_ and C_2_ are constants, and the E_i_ function is exponential integral which is defined as follows:

11 $$E_{i} \left( { - \chi } \right) = - \, \int \limits_{\chi }^{\infty } \frac{{e^{ - u} }}{u}du$$

Applying the initial condition (Eq. 7) or outer boundary condition (Eq. 9), the following result is obtained:

12 $$\left( {{\text{ P}}_{{\text{D}}} = 0{ }} \right){ }@{ }\left\{ {\begin{array}{*{20}c} {B.C 2{:} \, \left( {{\text{ r}}_{{{\text{De}}}} \to { }\infty } \right) } \\ {or} \\ { I.C{:} \, \left( {{\text{t}}_{{\text{D}}} \to { }0} \right) } \\ \end{array} } \right\}{ } \Rightarrow { }\left( {{ }{\mathbf{C}}_{2} = 0{ }} \right)$$

Thus, the Eq. (10) will be converted to a more simplified general solution to the diffusivity equation (Eq. 6), as follow:

13 $$P_{D} = C_{1} \cdot Ei\left( { - \frac{{r_{D}^{2} }}{{4t_{D} }}} \right)$$

With the imposing of the inner boundary condition (Eq. 8), the constant coefficient, ${C}_{1}$, can be obtained.

when ${r}_{D}\to 1$, we obtain:

14 $$1 = C_{1} \cdot Ei\left( { - \frac{1}{{4t_{D} }}} \right)$$

As a result, a constant value is not obtained for **C**_**1**_.

Although the combination of variable method could not provide an exact analytical solution for the diffusivity equation (regarding initial and all boundary conditions) considered in this study; it provided a general format as a clue to estimate an approximate correlation. So, based on the above-mentioned general format as well as discrete data, an optimization algorithm was implemented, where an approximate empirical correlation was developed for dimensionless pressure as a function of ${r}_{D}$ and ${t}_{D}$. Therefore, based on the above-mentioned general format as well as discrete data, an optimization algorithm was implemented, so that, the keyword of “*FindFit*” is used in *Mathematica software* to find numerical values of the proposed function to best fit with the pressure data points obtained from the inverse of the Laplace transform of Van Everdingen and Hurst’s formula. As a result, an approximate empirical correlation was developed for dimensionless pressure as a function of ${r}_{D}$ and ${t}_{D}$. For more details about the optimization algorithm please refer to Appendix A.

The dimensionless pressure function would be provided as follows:

15 $${\text{P}}_{{\text{D}}} \left( {{\text{r}}_{{\text{D}}} ,{\text{t}}_{{\text{D}}} } \right) = \underbrace {{\frac{{({\text{r}}_{{\text{D}}} )^{{{\mathbf{f}}_{1} \left( {{\mathbf{r}}_{{\mathbf{D}}} ,{\mathbf{t}}_{{\mathbf{D}}} } \right)}} }}{{{\text{E}}_{{\text{i}}} \left( {\frac{ - 1}{{4{\text{t}}_{{\text{D}}} }}} \right)}}}}_{{{\varvec{C}}1}}{ } \times {\text{E}}_{{\text{i}}} \left( {\frac{{ - {\text{r}}_{{\text{D}}}^{2} }}{{4{\text{t}}_{{\text{D}}} }}} \right)$$

where

16 $${\mathbf{f}}_{1} \left( {{\mathbf{r}}_{{\mathbf{D}}} ,{\mathbf{t}}_{{\mathbf{D}}} } \right) = {\text{b}}\left( {{\text{t}}_{D} } \right) \times {\text{r}}_{{\text{D}}}^{{{\text{a}}\left( {t_{D} } \right)}}$$

17 $$a\left({t}_{D}\right) = \frac{1}{{\left( {2.573 + 6.576 \times 10^{ - 5} \times {\text{ t}}_{{\text{D}}}^{{7.910*10^{ - 1} }} } \right)}}$$

18 $$b\left({t}_{D}\right)=0.001+\frac{0.420}{{t}_{D}^{0.605}}$$

Equation (15) satisfies all the initial and boundary conditions as assumed from Eqs. (7) to (9).

For instance, for inner boundary condition when ${r}_{D}\to 1$, we obtain:

19 $${P}_{D}({r}_{D}=1,{t}_{D})=\frac{{(1)}^{{f}_{1}\left(1,{t}_{D}\right)}}{{E}_{i}(\frac{-1}{4{t}_{D}})} \times {E}_{i}\left(\frac{-{1}^{2}}{{4t}_{D}}\right)=1$$

**Figure 1:**
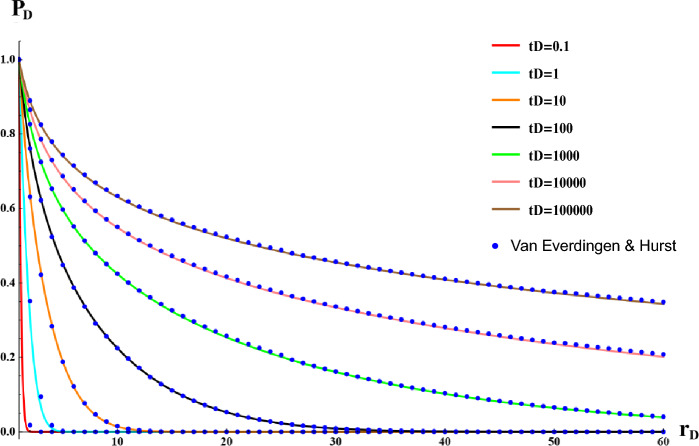
Comparison of the proposed function for dimensionless wellbore pressure with Van-Hurst numerical method. (Points indicate the data obtained from Van Everdingen and Hurst numerical method and solid lines are representative of the proposed method).

Evaluating the proposed function of dimensionless pressure is illustrated in Fig. 1. Results show a perfect agreement between the proposed method (15) and numerical method^4^.

**Figure 2:**
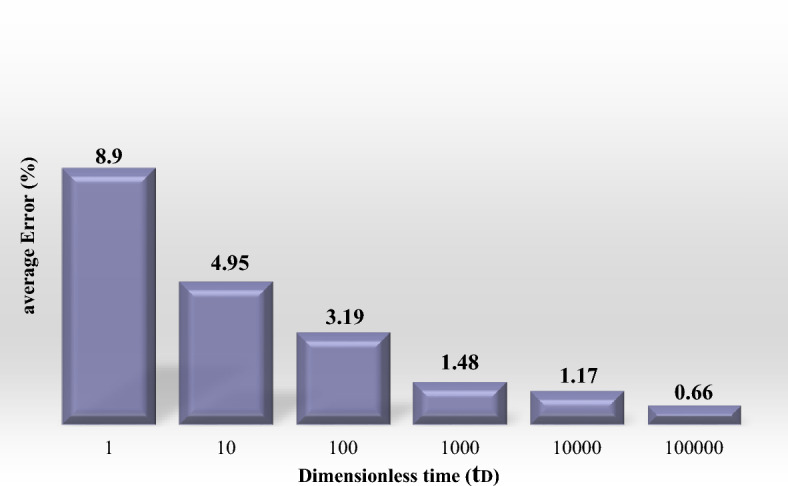
The bar chart of the average error of the proposed function compared to Van Everdingen and Hurst’s method for wellbore pressure.

For a vivid evaluation on the wellbore pressure function performance at different times, the bar chart of the average error between proposed function and Van Everdingen and Hurst method is presented by percentage in Fig. 2.

The pressure distribution is the most fundamental function in reservoir engineering which could be exerted in all studies pertinent to production at a constant wellbore pressure; flow rate function is a case in point.

Following conventional equation can be used to calculate the production rate:

20 $${\text{q}}\left( {\text{t}} \right) = \frac{{{\text{k}}\left( {2\uppi {\text{rh}}} \right)}}{\upmu }\left. {\frac{{\partial {\text{P}}}}{{\partial {\text{r}}}}} \right|_{{{\text{r}}_{{\text{w}}} }}$$

The dimensionless form of (20) for case of constant wellbore pressure would be as follows:

21 $${\text{q}}_{{\text{D}}} \left( {{\text{t}}_{{\text{D}}} } \right)=-{\mathrm{ r}}_{\mathrm{D}}{\left.\frac{\partial {\mathrm{P}}_{\mathrm{D}}}{\partial {\mathrm{r}}_{\mathrm{D}}}\right|}_{{\mathrm{ r}}_{\mathrm{D}}=1}$$

Inserting (15) in to the (21), finally, the dimensionless production rate would be achieved as follows:

22 $${\text{q}}_{{\text{D}}} \left( {{\text{t}}_{{\text{D}}} } \right) = - \left( {{\text{b}}\left( {{\text{t}}_{D} } \right) + \frac{{2{\text{e}}^{{ - \frac{1}{{4{\text{t}}_{{\text{D}}} }}}} }}{{{\text{Ei}}\left[ { - \frac{1}{{4{\text{t}}_{{\text{D}}} }}} \right]}}{ }} \right)$$

**Figure 3:**
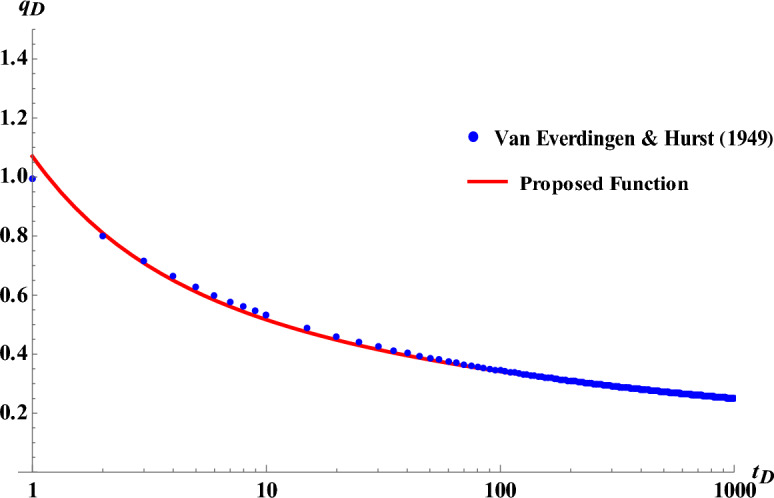
Comparison of the proposed function for dimensionless flow rate with numerical method^4^. (Points indicate the data obtained from Van Everdingen–Hurst numerical method and solid lines are representative of the proposed method).

Evaluating the proposed function for dimensionless flow rate is illustrated in Fig. 3. Result shows a perfect agreement between the proposed method (22) and numerical method^4^.

### Error investigation

Because of the importance of the flow rate function in pressure build up and wellbore storage calculations, the accuracy of the proposed function in early time is investigated through the comparison with two main studies in the literature.

**Figure 4:**
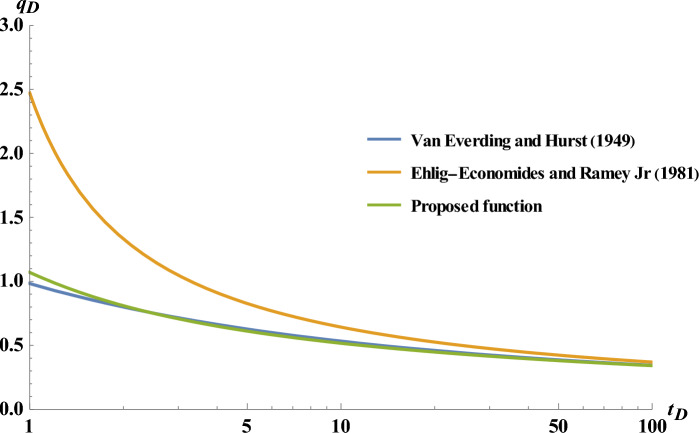
Comparison of the proposed flow rate fucntion with Ehlig-Economides and Ramey^8^ and Van Everdingen and Hurst^4^ method.

The quantitative comparison has been performed between the rate function obtained from this study and Van Everdingen and Hurst^4^ as well as Ehlig-Economides and Ramey^8^ indicated in the semi-log plot in Fig. 4.

As illustrated in Fig. 4, at early times, the results obtained from the proposed study is considerably superior to Economides’s approximate function, in comparison with exact numerical solution (as provided by Van Everdingen and Hurst^4^).

**Figure 5:**
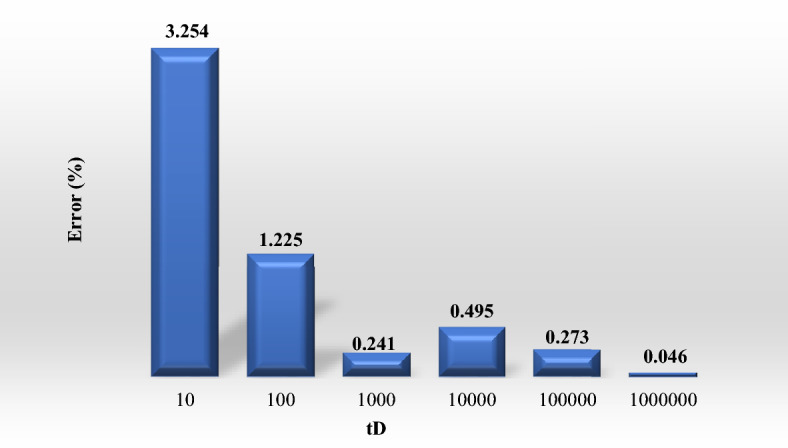
The bar chart of the relative error of the proposed function versus time in comparison to Van Everdingen and Hurst method.

For a clearer evaluation on the rate function performance at different times, the bar chart of the relative error between proposed function and Van Everdingen and Hurst method is presented by percentage in Fig. 5.

Moreover, results of calculating the average error at different intervals, are also provided in detail in Table 2 as follows:

**Table 2:** Average error for the proposed flow rate function, considering time domains.

Time interval	Average error (%)
10 < tD < 100	2.2
100 < tD < 10^6^	0.45

According to the information provided in Table 2, the rate function results in a negligible error at t_D_ > 100 (less than 0.5%), hence it can be used accurately for modeling the wellbore storage effect at early times.

It should be mentioned that for *tight* reservoirs because of ultralow formation permeability (Nano-Darcy range), even after several months of production, t_D_ will not reach to the value of ${10}^{6}$. As a result, the proposed function (22) is more applicable in predicting the flow rate in comparison with Ehlig-Economides and Ramey^8^**.**

## Calculation of pressure buildup following the production at a constant-bottom hole pressure

**Figure 6:**
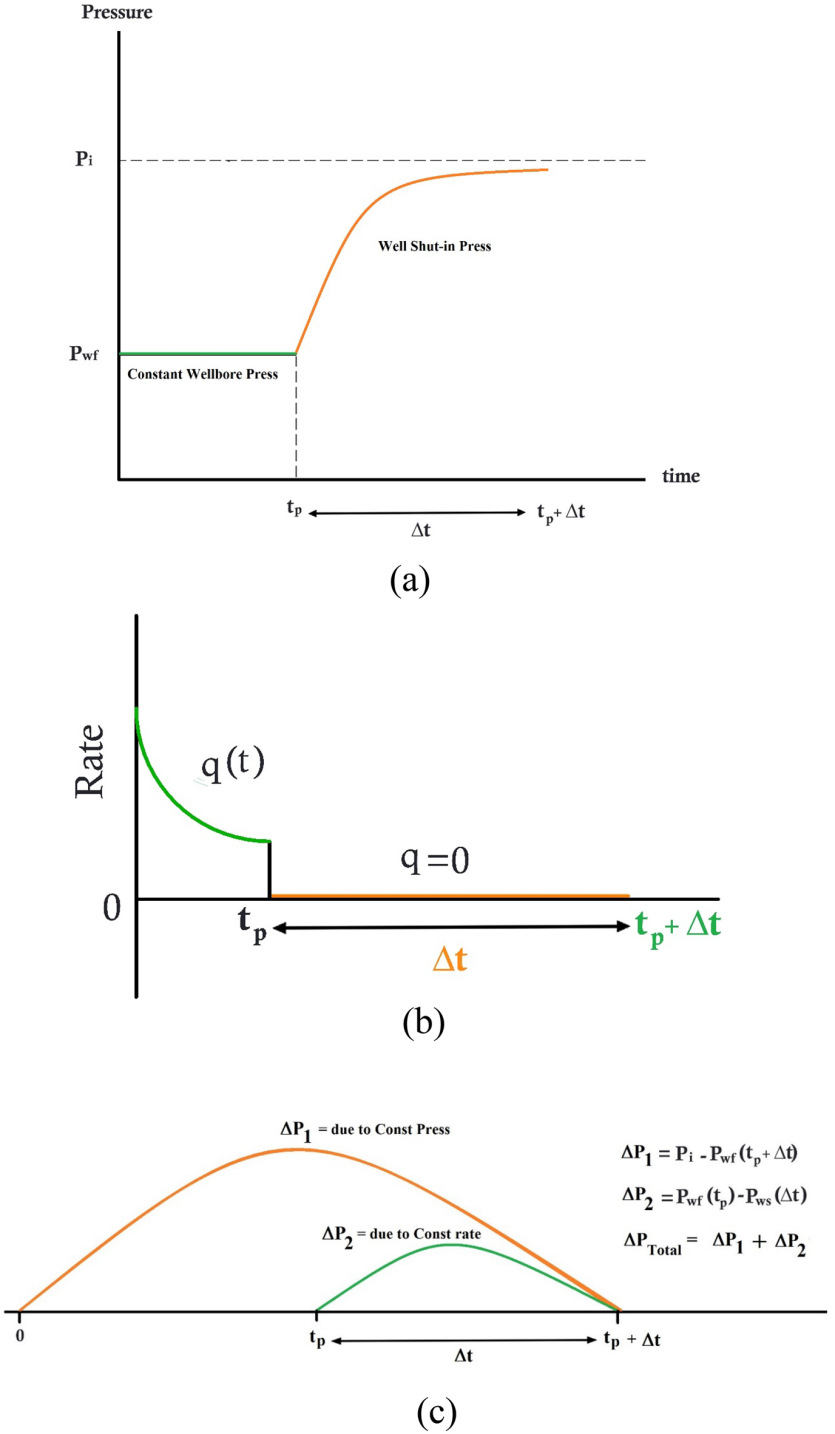
Schematic diagrams for pressure build up following constant wellbore pressure well test (a) pressure well shut-in (b) flow rate and (c) superposition pressure in pressure build-up test following constant wellbore pressure production.

In the pressure build-up test, the well is shut-in after production at a constant wellbore pressure during t_p_, and conditions turned into production at a constant rate (equals to zero). Schematic diagrams to show the wellbore pressure and rate before and after well shut-in are presented in Fig. 6 part (a) and (b) respectively.

According to Fig. 6c, the superposition principle is used to obtain well shut-in pressure ${P}_{ws}$ at time t = t_p_ + Δt:

23 $$\Delta {\text{P}}_{{{\text{Total}}}} = {\text{P}}_{{\text{i}}} - {\text{P}}_{{{\text{ws}}}} \left( {{\text{t}}_{{\text{P}}} + \Delta {\text{t}}} \right) = \Delta {\text{P}}_{{{\text{due }}\,{\text{to}} \,{\text{Const}} \,{\text{Press}}}} + \Delta {\text{P}}_{{{\text{due}}\,{\text{ to}} \,{\text{Const}} \,{\text{rate}} \left( {{\text{q}} = 0} \right) }}$$

To implement the principle of superposition, first it is assumed that the constant-pressure mode has uninterruptedly been continued up to time $\left({t}_{P}+\Delta t\right)$. Hence, the wellbore pressure drop due to constant pressure production is given by:

24 $$\Delta {\text{P}}_{{{\text{due }}\,{\text{to}} \,{\text{Const}} \,{\text{Press}}}}={P}_{i}-{P}_{wf} \left({t}_{P}+\Delta t\right)$$

Second, to simulate the zero-flow rate after time ${t}_{P}$ for shut-in mode, it is assumed that the same quantity of the corresponding flow rate of constant-pressure mode, has been injected to the reservoir between time ${t}_{P}$ and $\Delta t$. Therefore, the summation of flowrate between time ${t}_{P}$ and $\Delta t$ will be zero.

Applying the convolution theory in time to a continuously varying rate $q\left(t\right)$, gives expression for wellbore pressure drop due to constant-rate production, which may be written and defined as follow:

25 $$\begin{aligned}\Delta {\text{P}}_{{{\text{due}}\,{\text{ to}} \,{\text{Const}} \,{\text{rate}} \left( {{\text{q}} = 0} \right) }} & = {\text{P}}_{{{\text{wf}}}} { }\left( {{\text{t}}_{{\text{P}}} } \right) - {\text{P}}_{{{\text{ws}}}} { }\left( {{\Delta t}} \right) \\ & = \frac{\mu }{2\pi kh} \int \limits_{{t_{P} }}^{{t_{P} + \Delta t}} \left( { - q \left( \tau \right)} \right) \frac{{dP_{wD} }}{dt}\left( {t_{P} + \Delta t - \tau } \right) d\tau \\ \end{aligned}$$

where ${P}_{wD}$ is the dimensionless wellbore pressure drop for constant-rate production and $q\left(\tau \right)$ is the flowrate in constant pressure production.

By considering the above discussion, the negative sign for flow rate (−$\left(\tau \right)$) in Eq. (25) shows that the injection flowrate is assigned to reservoir between time ${t}_{P}$ and $\Delta t$ under constant-pressure production.

Substituting Eqs. (24) and (25) into Eq. (23) results in the following expressions for total wellbore pressure drop, given by:

26 $$P_{i} - P_{ws} \left( {t_{P} + \Delta t} \right) = P_{i} - P_{wf} \left( {t_{P} + \Delta t} \right) + \frac{\mu }{2\pi kh} \int \limits_{{t_{P} }}^{{t_{P} + \Delta t}} \left( { - q\left( \tau \right)} \right) \cdot \frac{{dP_{wD} }}{dt}\left( {t_{P} + \Delta t - \tau } \right) d\tau$$

Implementing some mathematical manipulations and using the dimensionless flowrate formula as defined in Eq. (22) eventuates in the following expression for the dimensionless form of Eq. (26), given by:

27 $$\frac{{P_{ws} \left( {t_{P} + \Delta t} \right) - P_{wf} \left( {t_{P} + \Delta t} \right)}}{{P_{i} - P_{wf} \left( {t_{P} + \Delta t} \right)}} = \int \limits_{{t_{PD} }}^{{t_{PD} + \Delta t_{D} }} q_{D} \left( \tau \right) \cdot \frac{{dP_{wD} }}{{dt_{D} }}\left( {t_{PD} + \Delta t_{D} - \tau } \right) d\tau$$

Equation (27) may be simplified and written as follow:

28 $$P_{D - WS} = \int \limits_{{t_{PD} }}^{{t_{PD} + \Delta t_{D} }} q_{D} \left( \tau \right) \cdot \frac{{dP_{wD} }}{{dt_{D} }}\left( {t_{PD} + \Delta t_{D} - \tau } \right) d\tau$$

where the dimensionless pressure for shut-in case, could be defined by the following format:

29 $$P_{D - WS} = \frac{{P_{ws} \left( {t_{P} + \Delta t} \right) - P_{wf} \left( {t_{P} + \Delta t} \right)}}{{P_{i} - P_{wf} \left( {t_{P} + \Delta t} \right)}}$$

here it should be noted that ${P}_{wf}$ at time ${t}_{P}+\Delta t$ is equal to ${P}_{wf}$ at time ${t}_{P}$ in all aforementioned equations.

Now, we recall the proposed flow rate function which was obtained and introduced in the section “Analysis”, for the transient-rate production (${q}_{D}\left(\tau \right)$), based on Eq. (22) in the context of manuscript.

Moreover, we use the exact analytical solution of constant-rate production as introduced by Matthews and Russell^14^ for pressure profile ($${P}_{Dw})$$ and the corresponding time derivative, as follows:

30 $$P_{D} = - \frac{1}{2}Ei\left( { - \user2{ }\frac{{r_{D}^{2} }}{{4 t_{D} }}} \right) \to P_{Dw} = - \frac{1}{2}Ei\left( { - \user2{ }\frac{1}{{4 t_{D} }}} \right) \to \frac{{{\varvec{dP}}_{{{\varvec{wD}}}} }}{{{\varvec{dt}}_{{\varvec{D}}} }} = \frac{{{\varvec{e}}^{{ - \user2{ }\frac{1}{{4\user2{ t}_{{\varvec{D}}} }}}} }}{{2\user2{ t}_{{\varvec{D}}} }}$$

Substituting Eqs. (30) and (22) into Eq. (28), pressure build-up is calculated exactly from the following integral function:

31 $$P_{D - WS} = \int \limits_{{t_{PD} }}^{{t_{PD} + \Delta t_{D} }} - \left( {b\left( \tau \right) + \frac{{2{\text{e}}^{{ - \frac{1}{4*\tau }}} }}{{Ei\left[ { - \frac{1}{4*\tau }} \right]}}} \right) \cdot \frac{{e^{{ - \frac{1}{{4 (t_{PD} + \Delta t_{D} - \tau )}}}} }}{{2 (t_{PD} + \Delta t_{D} - \tau )}} d\tau$$

**Figure 7:**
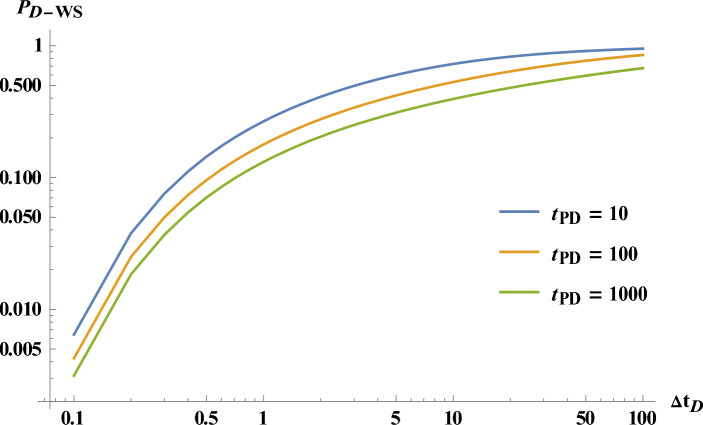
Dimensionless well pressure in case of well shut-in pressure for different tp.

Figure 7 shows the well shut-in pressure.

It should be noted that the mathematical expressions and modeling approach rendered for the above-mentioned case (*Pressure Buildup Following the Production at a Constant-Bottom Hole Pressure)* is the basis of our equations and foundation for the current study, which then in a special way would be developed in section “Calculation of pressure buildup following the production at a constant well head pressure” for the case of pressure buildup test following the production at a constant well head pressure to include wellbore storage effect.

### Comparison with field data/constant bottom hole pressure

We extracted real field data from Economides’ study^8^ which is as follows:

A well was produced at a constant bottom hole pressure. At the time of shut-in, fluid properties and formation properties presented respectively in Tables 3 and 4 as follows:

**Table 3:** Fluid properties for buildup test example.

$\mu$	*B*	*C*_*t*_	*r*_*w*_	*h*	*k*	$\varnothing$	*P*_*wf*_
65 cp	1.2 res bbl/STB	$15\times {10}^{-6}$ Psi^−1^	0.33 ft	130 ft	92.5 md	0.23	41 Psig

**Table 4:** Formation properties for buildup test example.

$\Delta t$ (h)	*P*_*ws*_ (Psig)
0	41
0.1	75
0.25	110
0.5	112
1	202
2	249
3	272
5	295
7	302
10	310
20	319
30	330

**Figure 8:**
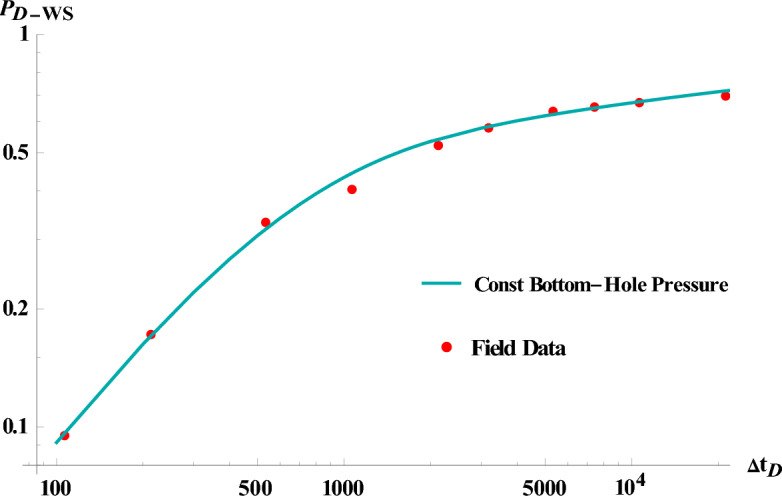
Verification the model (at constant bottom-hole pressure) with Field data.

Figure 8 depicts the dimensionless well shut-in pressure versus time in log–log plot, where an excellent agreement with field data is observed in this case.

It should be noted that, for the sake of fair comparison with real field data, the wellbore storage effect was applied in the modeling here. The wellbore storage effect in this condition is merely related to the closed time period of the well (shut-in well/during pressure build-up test), with no inclusion during the production period with constant bottom hole pressure.

Therefore, P_WD_ in the integral formula of Eq. (28) (*which is the dimensionless pressure of production at a constant rate*) is replaced from Eq. (53) to consider the wellbore storage effect which exclusively belongs to the duration of production at a constant rate. This equation and the related correlations will be later presented and fully discussed in section “Calculation of pressure buildup following the production at a constant well head pressure”.

### Comparison with eclipse numerical simulation/constant bottom hole pressure

In the simulated build up test, the well first produced at a constant wellbore pressure (4000psi) for three days, and then was shut-in (zero rate) for three days.

**Figure 9:**
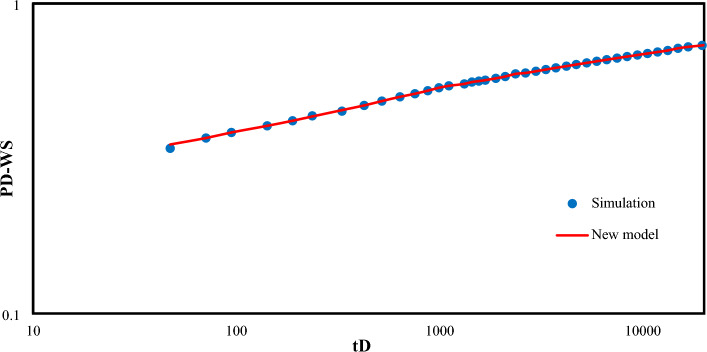
Comparison of the proposed function for dimensionless well shut-in pressure with numerical simulation result.

Figure 9 depicts the dimensionless well shut-in pressure versus time, where an excellent agreement with numerical simulation result is also observed in this case.

## Wellbore storage effect

The main objective of developing an approximate correlation is to develop a more applicable correlation which could be easily incorporated in complicated calculations, especially at early times. Considering the fact that the proposed correlation is able to predict the flow rate precisely at early times (as noted in Fig. 4), modeling of wellbore storage effect can also be possible for constant wellhead pressure production as well as pressure build up tests.

### Calculation of flow rate for production with constant pressure at the wellhead

During the production at a constant wellhead pressure, with the changes in the friction flow, the pressure drop at the wellbore varies as a function of the rate; therefore, the wellbore pressure is not constant ($\mathrm{P}\left({\mathrm{r}}_{\mathrm{w}},\mathrm{ t}\right)\ne cte)$.

Using the energy balance equation, ignoring thermal energy loss, incompressible, and considering laminar flow in the well, following equation is obtained:

32 $$P\left({r}_{w},t\right)={P}_{tf}+\frac{64Lq(t)\mu }{2{g}_{c}\pi {D}^{4}}+H\overline{\rho }$$

where $P\left({r}_{w},t\right)$ is the wellbore flowing pressure, $q(t)$ is the flow rate, ${P}_{tf}$ is the wellhead flowing pressure, $\overline{\rho }$ is the average fluid density in the wellbore, $L$ is the wellbore length, $D$ is the wellbore diameter and $H$ is the wellbore vertical length.

In an attempt of modeling constant wellhead pressure production, new dimensionless pressure and rate are defined as follows:

33 $${P}_{D}\left({r}_{D},{t}_{D}\right)=\frac{[{P}_{i}-P\left(r,{t}_{D}\right)]}{({P}_{i}-{P}_{tf}-{\text{b}}^{\prime })}$$

34 $${q}_{D}\left({t}_{D}\right)=\frac{q\left(t\right)\mu }{2\pi kh\left({P}_{i}-{P}_{tf}-{\text{b}}^{\prime }\right)}$$

where ${q}_{D}\left({t}_{D}\right)$ is the dimensionless flow rate in case of producing at well head constant pressure and $h$ is the thickness of formation.

Inserting (33) and (34) in (32), Eq. (35) is obtained as:

35 $${\text{P}}\left( {{\text{r}}_{{\text{w}}} , {\text{t}}} \right) = {\text{P}}_{{{\text{tf}}}} + {\text{a}}^{\prime } \left( {{\text{P}}_{{\text{i}}} - {\text{P}}_{{{\text{tf}}}} - {\text{b}}^{\prime } } \right){\text{q}}_{{\text{D}}} + {\text{b}}^{\prime }$$

The constant parameters $\mathrm{a{\prime}}$ and $\mathrm{b{\prime}}$ are a function of rock and fluid properties as well as well geometry, and are defined as follows:

36 $${\text{b}}^{\prime } = {\text{H}}\overline{\uprho }$$

37 $$a^{\prime} = \frac{64\,khL}{{g_{c} D^{4} }}$$

In production at a constant wellhead pressure, the wellbore storage effect is related to the inner boundary conditions using the conservation of mass equation.

The sheer production rate of the wellbore volume is as follows:

38 $${\text{q}}_{{\text{w}}} = \left( {\frac{{\partial {\text{v}}_{{\text{w}}} }}{{\partial {\text{t}}}}} \right) = - {\text{c}}_{{\text{w}}} {\text{v}}_{{\text{w}}} \frac{{\partial {\text{P}}_{{{\text{wf}}}} }}{{\partial {\text{t}}}}$$

where ${v}_{w}$ wellbore volume and ${\mathrm{c}}_{\mathrm{w}}$ is the wellbore fluid compressibility.

${v}_{w}$ includes the volume of the wellbore, the annulus, and any additional volume of fluid connected to the wellbore, which may be produced without changing the sand face pressure.

From mass balance, we have:

39 $${\text{q}}\left( {\text{t}} \right) = {\text{q}}_{{\text{w}}} + {\text{q}} = - {\text{v}}_{{\text{w}}} {\text{c}}_{{\text{w}}} \frac{{\partial {\text{P}}\left( {{\text{r}}_{{\text{w}}} ,{\text{t}}} \right)}}{{\partial {\text{t}}}} + \frac{{2\uppi {\text{kh}}}}{\upmu }\left( {{\text{r}}\frac{{\partial {\text{P}}}}{{\partial {\text{r}}}}} \right)_{{{\text{r}} = {\text{r}}_{{\text{w}}} }}$$

$\mathrm{q}(\mathrm{t})$ is the total surface fluid production rate, q_w_ is the sum of the production rate from the wellbore volume, and q is the production rate from the sand face.

Using (35):

40 $$\frac{{\partial {\text{P}}\left( {{\text{r}}_{{\text{w}}} ,{\text{t}}} \right)}}{{\partial {\text{t}}}} = \frac{{\partial {\text{P}}_{{{\text{tf}}}} }}{{\partial {\text{t}}}} + {\text{a}}^{\prime } \times \frac{{\partial \left( {{\text{P}}_{{\text{i}}} - {\text{P}}_{{{\text{tf}}}} - {\text{b}}^{\prime } } \right){\text{q}}_{{\text{D}}} }}{{\partial {\text{t}}}} + \frac{{\partial {\text{b}}^{\prime } }}{{\partial {\text{t}}}}$$

Inserting (40) into (39):

41 $$q\left( t \right) = - v_{w} c_{w} \left[ {\frac{{\partial P_{tf} }}{\partial t} + a^{\prime } \left( {P_{i} - P_{tf} - b^{\prime } } \right)\frac{{\partial q_{D} }}{\partial t}} \right] + \frac{2\pi kh}{\mu }\left( {r\frac{\partial P}{{\partial r}}} \right)_{{r = r_{w} }}$$

Non-dimensionalization based on the defined parameters (33), (34), (36) and (37) gives:

42 $${\text{q}}_{{\text{D}}} \left( {\text{t}} \right) = - {\text{C}}_{{\text{D}}} \left[ {\frac{{\partial {\text{P}}_{{{\text{tfD}}}} }}{{\partial {\text{t}}_{{\text{D}}} }} + {\text{a}}^{\prime } \frac{{\partial {\text{q}}_{{\text{D}}} }}{{\partial {\text{t}}_{{\text{D}}} }}} \right] - { }\left( {{\text{r}}_{{\text{D}}} \frac{{\partial {\text{P}}_{{\text{D}}} }}{{\partial {\text{r}}_{{\text{D}}} }}} \right)_{{{\text{r}}_{{\text{D}}} = 1}}$$

The dimensionless wellbore storage coefficient is defined as (43):

43 $$C_{D} = \frac{{v_{w} c_{w} }}{{2\pi h\emptyset c_{t} r_{w}^{2} }}$$

Since the wellhead pressure is constant, $\frac{\partial {P}_{tfD}}{\partial {t}_{D}}$ in (42) equals zero, with the definition of dimensionless production rate, (42) is simplified as follows:

44 $${\text{q}}_{{\text{D}}} \left( {{\text{t}}_{{\text{D}}} } \right) = - {\text{C}}_{{\text{D}}} \times {\text{a}}^{\prime } \times {\text{q}}_{{\text{D}}}^{\prime } + \left. {{\text{q}}_{{\text{D}}} } \right]_{{{\text{C}}_{{{\text{D}} = 0}} }}$$

Equation (44) is a first order linear differential equation. By solving (44) (based on method presented in Appendix B), we obtain (45):

45 $${\text{q}}_{{\text{D}}} \left( {{\text{t}}_{{\text{D}}} } \right) = \left( {\left[ { \int \limits_{{{\text{t}}_{{\text{D}}} \to 0}}^{{{\text{t}}_{{\text{D}}} }} {\text{e}}^{{\frac{{{\text{t}}_{{\text{D}}} }}{{{\text{C}}_{{\text{D}}} {\text{a}}^{\prime } }}}} \times \left. {{\text{q}}_{{\text{D}}} } \right]_{{{\text{C}}_{{{\text{D}} = 0}} }} } \right] + \left. {{\text{q}}_{{\text{D}}} } \right]_{{{\text{t}}_{{\text{D}}} \to 0}} } \right){\text{e}}^{{\frac{{ - {\text{t}}_{{\text{D}}} }}{{{\text{C}}_{{\text{D}}} {\text{a}}^{\prime } }}}}$$

According to (45), a specific rate function should be first determined for production at a constant wellbore pressure (${\left.{q}_{D}\right]}_{{C}_{D=0}}$) to provide the results and respective diagrams.

**Figure 10:**
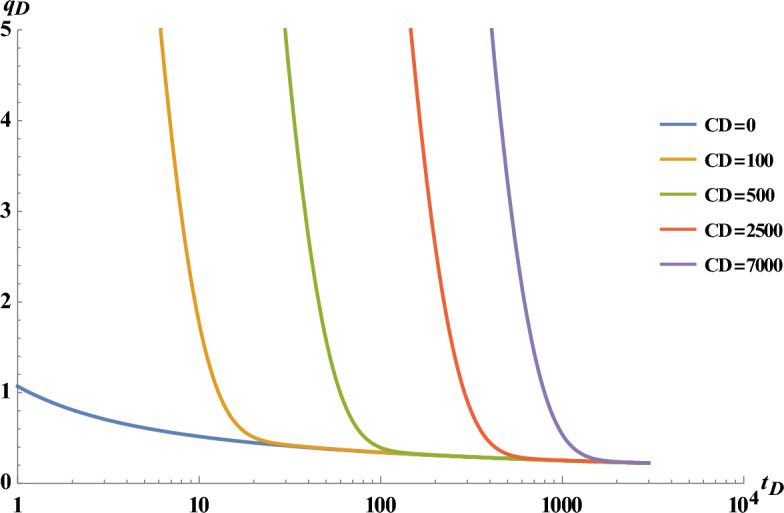
The effect of different wellbore storage coefficients on flow rate profiles.

Substituting (22) (for ${\left.{\mathrm{q}}_{\mathrm{D}}\right]}_{{\mathrm{C}}_{\mathrm{D}=0}}$) in (45), the effect of wellbore storage on the dimensionless rate for five different values of ${\mathrm{C}}_{\mathrm{D}}$ is presented in Fig. 10

As expected, the production rate shows difference only at early times. This difference appears when the rate was related to the wellbore volume. After a while, when the wellbore volume is filled, the production rate is only related to the reservoir rate, and independent of the well, therefore, the rates are equal whether the storage effect is considered or not (Fig. 10).

It should be noted that the main focus of the study is on wellbore storage effect, in an attempt of preventing any further side discussions, the concurrent effect of skin and wellbore storage are included and developed in Appendix C.

### Calculation of pressure buildup following the production at a constant well head pressure

Wellbore storage is caused by the fluid flowing into the wellbore after a well has been shut in on the surface, with the pressure in the wellbore changing as the height of the fluid in the wellbore changes.

In the pressure build-up test, the well is shut-in after production at a constant wellhead pressure during t_p_, and conditions turned into production at a constant rate (equal to zero). The superposition principle is used to obtain pressure at time t = t_p_ + Δt:

46 $$\Delta {\text{P}}_{{{\text{Total}}}} = {\text{P}}_{{\text{i}}} - {\text{P}}_{{{\text{ws}}}} \left( {{\text{t}}_{{\text{P}}} + \Delta {\text{t}}} \right) = \Delta {\text{P}}_{{{\text{due }}\,{\text{to}} \,{\text{Const}} \,{\text{Press}}}} + \Delta {\text{P}}_{{{\text{due}}\,{\text{ to}} \,{\text{Const}} \,{\text{rate}} \left( {{\text{q}} = 0} \right) }}$$

**Figure 11:**
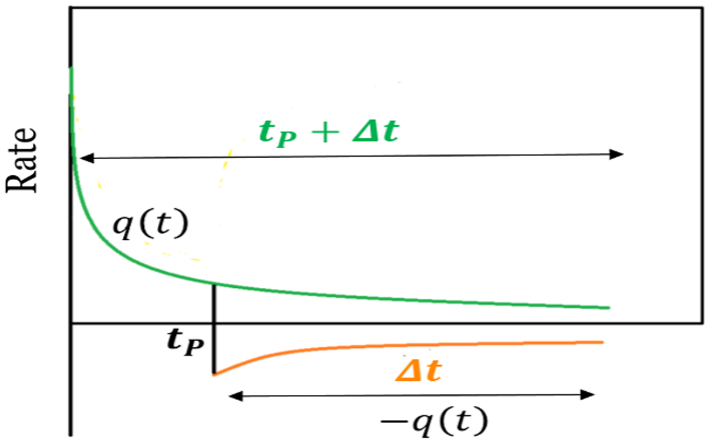
Scheme for modelling of superposition principle in pressure build-up test following constant well pressure production.

To implement the principle of superposition (as illustrated in Fig. 11), first it is assumed that the constant-pressure mode has uninterruptedly been continued up to time $\left({t}_{P}+\Delta t\right)$. Hence, the wellbore pressure drop due to constant pressure production, is given by Eq. (47):

47 $${\boldsymbol{\Delta} \mathbf{P}}_{{{\mathbf{due}}\, {\mathbf{to}} \,{\mathbf{Const}}\, {\mathbf{Press}}}}= P_{i} - P_{wf} { }\left( {t_{P} + \Delta t} \right)$$

Second, to simulate the zero-flow rate after time ${t}_{P}$ for shut-in state, it is assumed that the same quantity of the corresponding flow rate of constant-pressure mode, has been injected to the reservoir between time ${t}_{P}$ and $\Delta t$. Therefore, the summation of flowrate between time ${t}_{P}$ and ${t}_{P}+\Delta t$ would be zero.

To investigate the storage effect due to constant pressure, up to time $\left({t}_{P}+\Delta t\right)$, Eq. (48) representing the wellbore pressure, is used

48 $${\boldsymbol{\Delta} \mathbf{P}}_{{{\mathbf{due}}\, {\mathbf{to}} \,{\mathbf{Const}}\, {\mathbf{Press}}}} = {\text{P}}_{{\text{i}}} - {\text{P}}_{{{\text{wf}}}} \left( {{\text{t}}_{{\text{P}}} + {\Delta t}} \right) = {\text{ P}}_{{\text{i}}} - {\text{P}}_{{{\text{tf}}}} - {\text{a}}^{\prime } \left( {{\text{P}}_{{\text{i}}} - {\text{P}}_{{{\text{tf}}}} - {\text{b}}^{\prime } } \right){\text{q}}_{{\text{D}}} \left( {{\text{t}}_{{\text{P}}} + {\Delta t}} \right) - {\text{b}}^{\prime }$$

The pressure drop due to production with constant rate equal to zero or injection with negative flow rate ($-\mathrm{q}\left(\mathrm{t}\right)$), between time ${t}_{P}$ and ${t}_{P}+\Delta t$, with the application of the principle of superposition in time to a continuously varying rate $q\left(t\right)$, can be written and defined as follow.

49 $${\boldsymbol{\Delta} \mathbf{P}}_{{{\mathbf{due}} \,{\mathbf{to}} \,{\mathbf{Const}}\,{ }{\mathbf{rate }}\left( {{\mathbf{q = 0}}} \right)}} = \frac{\upmu }{{2\uppi {\text{kh}}}} \int \limits_{{{\text{t}}_{{\text{P}}} }}^{{{\text{t}}_{{\text{P}}} + \Delta {\text{t}}}} \left( { - {\text{q}}\left(\uptau \right)} \right) \frac{{ {\text{dP}}_{{{\text{wD}}}} }}{{{\text{dt}}}}\left( {{\text{t}}_{{\text{P}}} + \Delta {\text{t}} -\uptau } \right) {\text{d}}\uptau$$

where ${P}_{wD}$ is the dimensionless pressure for production at a constant rate and $q\left(\tau \right)$ is production rate at a constant wellbore pressure. Accordingly, the negative sign in $-q\left(\tau \right)$ indicates the injection process.

Substituting (48) and (49) in (46), ${{\varvec{\Delta}}\mathbf{P}}_{\mathbf{T}\mathbf{o}\mathbf{t}\mathbf{a}\mathbf{l}}$ will be obtained as Eq. (50):

50 $$\begin{aligned} {\boldsymbol{\Delta} \mathbf{P}}_{{{\mathbf{Total}}}} = & {\text{P}}_{{\text{i}}} - {\text{P}}_{{{\text{tf}}}} - {\text{a}}^{\prime } \left( {{\text{P}}_{{\text{i}}} - {\text{P}}_{{{\text{tf}}}} - {\text{b}}} \right)\times{\text{q}}_{{\text{D}}} \left( {{\text{t}}_{{\text{P}}} + {\Delta t}} \right) - {\text{b}}^{\prime } \\ & + \frac{\mu }{2\pi kh} \int \limits_{{t_{P} }}^{{t_{P} + \Delta t}} \left( { - q\left( \tau \right)} \right)\frac{{ dP_{wD} }}{dt}\left( {t_{P} + \Delta t - \tau } \right) d\tau \\ \end{aligned}$$

With considering the wellbore storage effect and well shut-in, the dimensionless pressure in the well is defined as (51):

51 $${\text{P}}_{{{\text{D}} - {\text{WS}}}} = \frac{{{\text{P}}_{{{\text{ws}}}} \left( {{\text{t}}_{{\text{P}}} + {\Delta t}} \right) - {\text{P}}_{{{\text{tf}}}} - {\text{b}}^{\prime } }}{{{\text{P}}_{{\text{i}}} - {\text{P}}_{{{\text{tf}}}} - {\text{b}}^{\prime } { }}}$$

Equation (50) is rewritten by considering Eq. (51) and implementing simplification and non-dimensionalization as follows:

52 $${\mathbf{P}}_{{{\mathbf{D}} - {\mathbf{WS}}}} = {\text{a}}^{\prime } {\text{q}}_{{\text{D}}} \left( {{\text{t}}_{{\text{P}}} + \Delta {\text{t}}} \right) - \int \limits_{{{\text{t}}_{{\text{P}}} }}^{{{\text{t}}_{{\text{P}}} + \Delta {\text{t}}}} { }\left( { - {\text{q}}_{{\text{D}}} \left(\uptau \right)} \right)\frac{{{\text{ dP}}_{{{\text{wD}}}} }}{{{\text{dt}}}}\left( {{\text{t}}_{{{\text{PD}}}} + \Delta {\text{t}}_{{\text{D}}} -\uptau } \right){\text{ d}}\uptau$$

Equation (52) is a complex equation and could not be used in the pressure build-up integral. Furthermore, the wellbore storage effect is eliminated far before the well shut-in (${\mathrm{t}}_{\mathrm{PD}}$). Thus for simplification, ${\mathrm{q}}_{\mathrm{D}}$ for production at a constant wellbore pressure (${\mathrm{C}}_{\mathrm{D}=0}$ (22)) is alternated with ${\mathrm{q}}_{\mathrm{D}}$ which is obtained from Eq. (45) (based on the results provided in Fig. 10 and related descriptions).

${P}_{wD}$ is the dimensionless pressure of production at a constant rate by considering the wellbore storage effect. ${P}_{wD}$ in the form of an integral function, with applying the *Millens formula* and *inverse Laplace* equation can be presented as follow:

53 $${\text{P}}_{{{\text{wD}}}} \left( {{\text{t}}_{{\text{D}}} } \right) = \int \limits_{0}^{\infty } \frac{{\left( {1 - {\text{e}}^{{ - {\text{u}}^{2} {\text{t}}_{{\text{D}}} }} } \right){\text{J}}_{0} \left( {\text{u}} \right){\text{du}}}}{{{\text{u}}\left( {\left( {1 + {\text{u}}^{2} {\text{C}}_{{\text{D}}} \frac{{\uppi }}{2}{\text{Y}}_{0} \left( {\text{u}} \right)} \right)^{2} + \left( {{\text{u}}^{2} {\text{C}}_{{\text{D}}} \frac{{\uppi }}{2}{\text{J}}_{0} \left( {\text{u}} \right)} \right)^{2} } \right)}}$$

where $u$ is the variable of integration, ${C}_{D}$ is the dimensionless wellbore storage, and ${J}_{0}$ and ${Y}_{0}$ are Bessel functions of zero order of first and second kind respectively.

Differentiating Eq. (53) results in:

54 $$\frac{{{\text{ dP}}_{{{\text{wD}}}} }}{{{\text{dt}_{{{\text{D}}}}}}} = \int \limits_{0}^{\infty } \frac{{ - \left( {{\text{u}}^{2} {\text{e}}^{{ - {\text{u}}^{2} {\text{t}}_{{\text{D}}} }} } \right){\text{J}}_{0} \left( {\text{u}} \right)}}{{{\text{u}}\left( {\left( {1 + {\text{u}}^{2} {\text{C}}_{{\text{D}}} \frac{{\uppi }}{2}{\text{Y}}_{0} \left( {\text{u}} \right)} \right)^{2} + \left( {{\text{u}}^{2} {\text{C}}_{{\text{D}}} \frac{{\uppi }}{2}{\text{J}}_{0} \left( {\text{u}} \right)} \right)^{2} } \right)}}$$

In order to calculate the well shut-in pressure, Eqs. (54) and (22) are inserted into (52).

**Figure 12:**
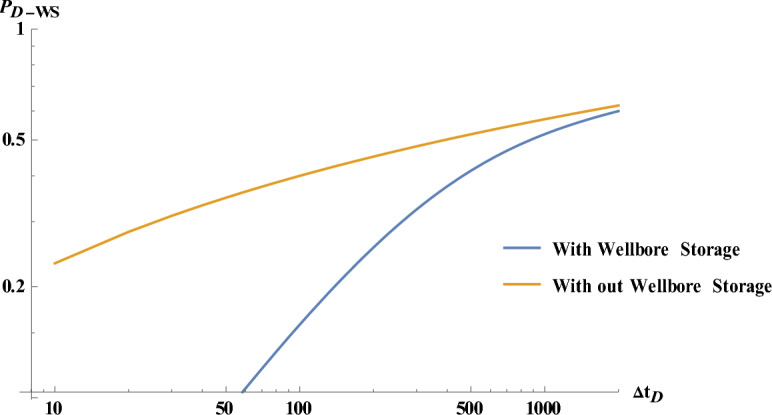
Comparison of the well pressure in case of well shut-in pressure, with and without considering wellbore storage effect.

Figure 12 compares the well shut-in pressure considering with and without wellbore storage effect.

The above results suggest that the proposed model (as implemented in Eq. 52) together with an appropriate sensitivity analysis, could be used to produce type curves, as a priori estimation tool, for analyzing pressure build-up field data of wells produced at constant wellhead pressure then shut-in with high accuracy while lowering the required costs and time of computation.

### Comparison with eclipse numerical simulation

In this part, commercial ECLIPSE software is employed for simulation and specified parameters for simulation model presented in Table 5. The results obtained from variable flow rate (due to production at a constant wellhead pressure) as well as pressure build-up test following production at a constant wellhead pressure, were examined.

**Table 5:** Specified parameters for simulation model.

Characteristic	Symbol	Value
Initial pressure	P_i_	5000 psi
Permeability	k	1 md
Porosity	Φ	0.2
Total compressibility	c_t_	2 × 10^–5^ psi^−1^
Well radius	r_w_	0.25 ft
Viscosity	µ	0.88 cp
Reservoir thickness	h	100 ft

#### Constant wellhead pressure (Transient rate decline)

To investigate the wellbore storage effect, THP (Tubing Head Pressure) should be set constant (constant wellhead pressure). Therefore, the wellhead pressure is set at 2000 psi.

The “WBOREVOL” keyword, which indicates the effective wellbore volume, is activated in ECLIPSE to determine the wellbore storage effect.

**Figure 13:**
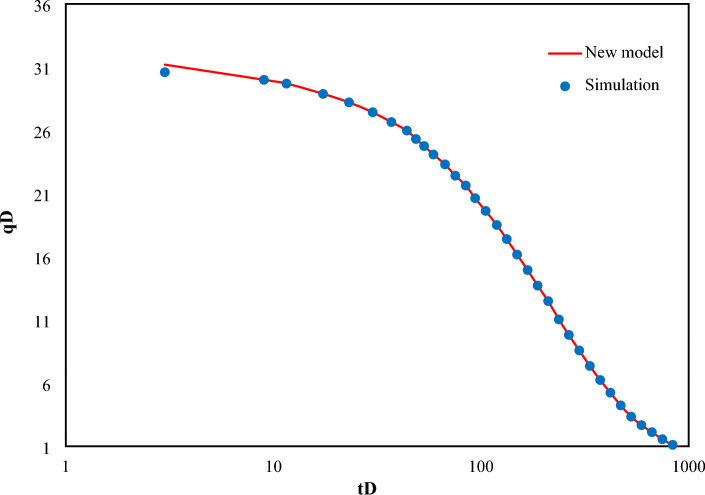
Comparison of the production rate in the constant wellhead pressure case with the simulation result.

Figure 13 indicates the obtained results from numerical simulation at ${C}_{D}=7000$ in comparison with the results from Eq. (45) (which was already represented in section “Calculation of flow rate for production with constant pressure at the wellhead” and illustrated in Fig. 10).

#### Pressure build-up following constant wellhead pressure

To run the simulation, wellhead pressure of 2000 psi is adjusted for period of production at a constant wellhead pressure, before a well has been shut in.

In addition to including the “WBOREVOL” keyword, the “STOP” keyword for shutting in the well from wellhead location, is also activated in ECLIPSE.

**Figure 14:**
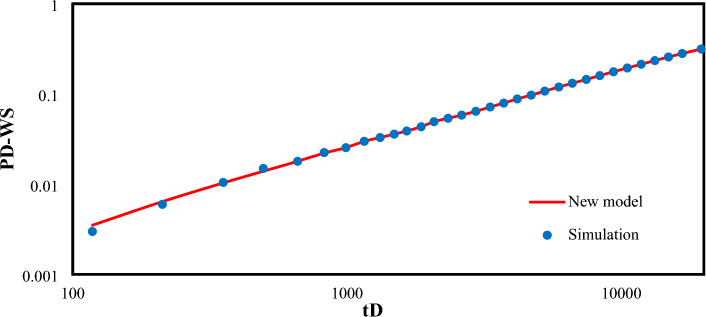
Comparison of the proposed function for well shut-in pressure with the simulation result.

Results obtained from numerical simulation at ${C}_{D}=7000$ are compared with the results from Eq. (52), and illustrated in Fig. 14.

Figures 13 and 14 represent excellent agreement between the results obtained from numerical simulation and the results in this study. This further confirms the reliability and outstanding performance of the modelling provided in this study.

## Conclusion

In this study, new models were presented for one-dimensional unsteady state fluid flow in an infinite-acting radial reservoir under constant pressure well-test operation. In the developed models, the effect of the wellbore storage was enumerated for the first time, which was commonly neglected in previous studies. This endeavor concentrated on modeling the two types of well-test operation; producing at constant wellhead pressure and pressure build-up following constant wellhead pressure production (constant pressure build-up).

It should be noted that given the wellbore storage effect at early times, a certain efficient rate function is required to be used at early times.

Considering the challenges ahead in the modeling process, the contribution of this study is fourfold:

- Proposing an approximate and efficient formula, for flow rate produced under constant bottom hole pressure in an infinite acting radial reservoir by implementing the multistep optimization process.
- Developing the mathematical differential equation based on the proposed function to include the wellbore storage effect for production under constant well head pressure.
- Developing a novel mathematical model for wellbore storage effect for pressure build-up following a constant wellhead pressure production by incorporating the proposed formula for flow rate.
- Validation of the obtained results with numerical simulation and field data of well-testing operations.

### Supplementary Information

Supplementary Information.

## Data Availability

All data generated or analyzed during this study are included in this published article. It will be available upon request. The corresponding author (MRK) should be contacted for this purpose.

## List of symbols

*A*: Area, L^2^
*C*_*t*_: Total compressibility, Lt^2^/m
*B*: Formation volume factor, dimensionless
*η*: $$\frac{k}{\phi \mu c}\,{\text{diffusivity}},{\text{L}}^{2} /{\text{t}}$$
*C*_*D*_: Dimensionless wellbore storage coefficient, $\frac{{\mathrm{v}}_{\mathrm{w}}{\mathrm{c}}_{\mathrm{w}}}{2\pi h\mathrm{\varnothing }{c}_{t}{r}_{w}^{2}}$
*D*: Wellbore diameter, L
*g*_*c*_: Units conversion factor
*h*: Formation thickness, L
*k*: Reservoir absolute permeability, L^2^
*L*: Wellbore length, L
*H*: Wellbore vertical length, L
*r*: Distance from the well, L
*N*_*p*_: Cumulative production, L^3^
*S′*: Storage coefficient of aquifer Lt^2^/m
*P*: Pressure, m/Lt^2^
$$v_{w}$$: Wellbore volume, L^3^
*P*_*i*_: Initial reservoir pressure, m/Lt^2^
*P*_*tf*_: Flowing wellhead pressure, m/Lt^2^
*P*_*wD*_: Dimensionless wellbore pressure, $\frac{\left(2\pi kh\right){(\mathrm{P}}_{\mathrm{i}}-{\mathrm{P}}_{\mathrm{w}})}{\mu q}$
*P*_*wf*_: Flowing bottom-hole pressure, m/Lt^2^
$S_{w}$: The drawdown of the well, m/Lt^2^
$l$: Laplace space variable
$\overline{{q_{D} }} \left( l \right)$: Laplace transform of dimensionless flow rate
$K_{0}$: Modified Bessel function of zero order of second kind
$u$: Integral variable in Mellin’s inversion formula
$Y_{0} \left( u \right)$: Bessel function of zero order of second kind
*q*: Flow rate, L^3^/t
$c_{w}$: Wellbore fluid compressibility Lt^2^/m
q_D_: Dimensionless flow rate, $\frac{\mu q\left(t\right)}{2\pi kh ({P}_{i}-{P}_{wf})}$
*r*_*D*_: Dimensionless radius, *r*/*r*_*w*_
*r*_*e*_: Reservoir radius,* L*
*r*_*eD*_: Dimensionless reservoir radius, *r*_*e*_*/r*_*w*_
*r*_*w*_: Wellbore radius, *L*
*t*: Time, *t*
*t*_*D*_: Dimensionless time, $$\frac{\mathrm{k}}{\mathrm{\phi \mu c}{\mathrm{ r}}_{\mathrm{w}}^{2}}$$
*t*_*DA*_: Dimensionless time based on drainage area
*t*_*p*_: Production time, *t*
*T*: Transmissibility, $$\frac{\mathrm{kh}}{\upmu }$$
*S*: Skin factor
$\mu$: Fluid viscosity, m/Lt
$$\emptyset$$: Porosity
$\overline{\rho }$: Average wellbore density, m/L^2^
$\tau$: Variable of integration
$u$: Variable of integration
*P*_*D*_: Dimensionless pressure ratio, $\frac{{\mathrm{P}}_{\mathrm{i}}-\mathrm{P}(\mathrm{r},\mathrm{t})}{{\mathrm{P}}_{\mathrm{i}}-{\mathrm{P}}_{\mathrm{w}}}$
*P*_*ws*_: Bottom-hole pressure after shut-in, m/Lt^2^
$Ei\left( { - \user2{ }{\text{x}}} \right)$: Exponential integral function
$\overline{{{\text{P}}_{{\text{D}}} }} \left( l \right)$: Laplace transform of dimensionless pressure
$K_{1}$: Modified Bessel function of first order of second kind
$J_{0} \left( u \right)$: Bessel function of zero order of first kind
