# Supplementary material for: Develop an empirical flow rate correlation to model wellbore storage phenomenon for wells produced at a constant wellhead pressure
Source: Sci Rep. 2023 Oct 18;13:17726. doi: 10.1038/s41598-023-44678-3 (PMC10584919; doi:10.1038/s41598-023-44678-3)
Supplement: Supplementary file 1 — Supplementary Information. [file 41598_2023_44678_MOESM1_ESM.docx]

**Appendix A**

**Optimization Algorithm process**

Now, again refer to the context of the manuscript and recall the general format of the proposed model for pressure (which is obtained in the manuscript) given as equation (13) as follows:

| $P_{D}=C_{1}.Ei\left( -\frac{r_{D}^{2}}{4t_{D}} \right)$ | (13) |
| --- | --- |

And also, the **inner** boundary condition is as follows:

| $\boldsymbol{B.C.\#1} P=P_{w} \boldsymbol{\to} \left. P_{D} \right\vert_{r_{D}=1}=1$ | (8) |
| --- | --- |

To obtain the constant coefficient, $C_{1}$, the **inner** boundary condition should be applied.

when $r_{D}\to1$, we obtain:

| $1=C_{1}.Ei\left( -\frac{1}{4t_{D}} \right)$ | (14) |
| --- | --- |

Or as a better depiction:

| $C_{1}=\frac{1}{Ei\left( -\frac{1}{4t_{D}} \right)}$ | (14) |
| --- | --- |

It means, if we suppose a **function** in the numerator of C1, this function must be **equal to 1** when $\boldsymbol{r}_{\boldsymbol{D}}\boldsymbol{\to1}$, to satisfy the situation of inner boundary condition.

Hence, the numerator may have a format of $({r_{D})}^{\mathbf{f}_{\mathbf{1}}\left( \mathbf{r}_{\mathbf{D}}\mathbf{,}\mathbf{t}_{\mathbf{D}} \right)}$, because of 1 power to anything will be equal to 1.

Therefore, by applying the inner boundary condition and considering the aforementioned general format for the numerator of C1, the following expression is achieved:

| $C_{1}=\underset{\boldsymbol{C}\boldsymbol{1}}{\underbrace{\frac{({r_{D})}^{\mathbf{f}_{\mathbf{1}}\left( \mathbf{r}_{\mathbf{D}}\mathbf{,}\mathbf{t}_{\mathbf{D}} \right)}}{E_{i}(\frac{-1}{4t_{D}})}}} \mathrm{when} r_{D}\to1 C_{1}=\frac{({1)}^{\mathbf{f}_{\mathbf{1}}\left( \mathbf{r}_{\mathbf{D}}\mathbf{,}\mathbf{t}_{\mathbf{D}} \right)}}{Ei\left( -\frac{1}{4t_{D}} \right)} \to C_{1}=\frac{(1)}{Ei\left( -\frac{1}{4t_{D}} \right)}$ | (15) |
| --- | --- |

Afterward, we used the keyword of "***FindFit***" in Mathematica software to find numerical values of the proposed function to **best fit** with the pressure data points obtained from the inverse of the Laplace transform of Van Everdingen & Hurst's formula.

This process continues until either of the goals specified by "PrecisionGoal" is achieved.

The possible setting for the method option for optimization in Mathematica includes "*ConjugateGradient*”, “*LevenbergMarquardt*”, “*Newton*”, “*QuasiNewton*", and “*NMinimize*", with the default being “Automatic".

It should be noted that, “Automatic" represents an option or other value that is to be chosen automatically by a built-in function.

Also, possible settings for the "*NMinimize*" option include "*Nelder Mead*”, “*Differential Evolution*", "*Simulated Annealing*", and "*Random Search*", which are well-known methods in mathematics.

**Appendix B**

**Solving first ODE for flow rate considering wellbore storage effect**

Given a first order non-homogeneous linear differential equation:

| $y^{/}+p\left( t \right)y=f(t)$ | ( 1) |
| --- | --- |

Follow these steps to determine the general solution $y(t)$ using an integrating factor:

1. Calculate the integrating factor $I\left( t \right)$.
2. Multiply the standard form equation by $I\left( t \right)$.
3. Simplify the left-hand side to

| $I\left( t \right)=e^{\int p\left( t \right)dt}$ | ( 2) |
| --- | --- |
| $\frac{d}{dt} [I\left( t \right)y]$ | ( 3) |

1. Integrate both sides of the equation.
2. Solve for $y(t)$.

The solution can be compactly written as:

| $y\left( t \right)=e^{-\int p\left( t \right)dt}[\int e^{\int p\left( t \right)dt} f\left( t \right)dt+C]$ | ( 4) |
| --- | --- |

Using the above method, we can re-write the equation (44) in the form of equation (1) as follows:

$$q_{D}^{/}+\frac{1}{C_{D}{\times a}^{/}} q_{D}=q_{D}|_{C_{D}=0} \times\frac{1}{C_{D}\times a^{/}}$$

Hence, we can find the solution easily like as equation (4):

$$q_{D}\left( t_{D} \right)=e^{\frac{-t_{D}}{C_{D}a}}'\left( \left[ \int_{t_{D}\to0}^{t_{D}} e^{\frac{t_{D}}{C_{D}a}}'\times\left. q_{D} \right]_{C_{D=0}} \right]+\left. q_{D} \right]_{t_{D}\to0} \right)$$

**Appendix C**

**Wellbore Storage and Skin Effect for Production Under Constant Pressure at The Well Head**

By considering the skin effect, the inner boundary condition for a well which produced at a constant pressure would be as follow:

| $P\left( r_{w},t \right)=P_{\mathrm{wf}}+S\left( r\frac{\partial P}{\partial r} \right)_{r=r_{w}}$ | (1) |
| --- | --- |

where S is the wellbore skin factor, and $P_{\mathrm{wf}}$ is the flowing bottom hole pressure.

During the production at a constant wellhead pressure, with the changes in the friction flow, the pressure drop at the wellbore varies as a function of the rate; therefore, the wellbore sand face pressure is not constant ($P\left( r_{w}.t \right)\neq cte)$.

The equation for the pressure drop in the wellbore for flowing liquid, using the energy balance equation, ignoring thermal energy loss, incompressible, and considering laminar flow in the well, is given by:

| $P_{\mathrm{wf}}=P_{\mathrm{tf}}+a'+b'$ | (2) |
| --- | --- |

Where, $P_{\mathrm{tf}}$ is the well head flowing pressure and the constant parameters $a'$ and $b'$ are a function of rock and fluid properties as well as well geometry, and are defined as follows:

| $b^{'}=H\bar{\rho}$ | (3) |
| --- | --- |
| $a^{'}=\frac{64khL}{g_{c}D^{4}}$ | (4) |

In an attempt of modeling constant wellhead pressure production, new dimensionless pressure and rate are defined as follows:

| $P_{D}\left( r_{D},t_{D} \right)=\frac{[P_{i}-P\left( r,t_{D} \right)]}{(P_{i}-P_{\mathrm{tf}}-b')}$ | (5) |
| --- | --- |
| $q_{D}\left( t_{D} \right)=\frac{q\left( t \right)\mu}{2\pi kh\left( P_{i}-P_{\mathrm{tf}}-b^{'} \right)}$ | (6) |

Inserting (33) and (34) in (32), equation (35) is obtained as:

| $P\left( r_{w},t \right)=P_{\mathrm{tf}}+a^{'}\left( P_{i}-P_{\mathrm{tf}}-b^{'} \right)q_{D}+b^{'}+S\left( r\frac{\partial P}{\partial r} \right)_{r=r_{w}}$ | (7) |
| --- | --- |

In production at a constant wellhead pressure, the wellbore storage effect is related to the inner boundary conditions using the conservation of mass equation.

The sheer production rate of the wellbore volume is as follows:

| $q_{w}=(\frac{\partial v_{w}}{\partial t})=-c_{w}v_{w}\frac{\partial P\left( r_{w},t \right)}{\partial t}$ | (8) |
| --- | --- |

$v_{w}$ includes the volume of the wellbore, the annulus, and any additional volume of fluid connected to the wellbore, which may be produced without changing the sand face pressure, q is the total surface fluid production rate, q_w_ is the sum of the production rate from the wellbore volume, and q is the production rate from the sand face. From mass balance, we have:

| $q\left( t \right)=q_{w}+q=-v_{w}c_{w}\frac{\partial P\left( r_{w},t \right)}{\partial t}+ \frac{2\pi kh}{\mu}\left( r\frac{\partial P}{\partial r} \right)_{r=r_{w}}$ | (9) |
| --- | --- |

Using (35):

| $\frac{\partial P\left( r_{w},t \right)}{\partial t}= \frac{\partial P_{\mathrm{tf}}}{\partial t}+a^{'}\frac{\partial\left( P_{i}-P_{\mathrm{tf}}-b^{'} \right)q_{D}}{\partial t}+ \frac{\partial b^{'}}{\partial t}+S \frac{\partial\left( r\frac{\partial P}{\partial r} \right)_{r=r_{w}}}{\partial t}$ |  | (10) |
| --- | --- | --- |

Inserting (40) into (39):

| $q\left( t \right)=-v_{w}c_{w}\left[ \frac{\partial P_{\mathrm{tf}}}{\partial t}+a^{'}\left( P_{i}-P_{\mathrm{tf}}-b^{'} \right)\frac{{\partial q}_{D}}{\partial t}+S \frac{\partial\left( r\frac{\partial P}{\partial r} \right)_{r=r_{w}}}{\partial t} \right]+ \frac{2\pi kh}{\mu}\left( r\frac{\partial P}{\partial r} \right)_{r=r_{w}}$ | (11) |
| --- | --- |

Non-dimensionalization based on the defined parameters ((33), (34),(36) and (37)) gives:

| $q_{D}\left( t \right)=-C_{D}\left[ \frac{\partial P_{\mathrm{tfD}}}{\partial t_{D}}+a^{'}\frac{{\partial q}_{D}}{\partial t_{D}}+S\frac{{\partial q}_{D}}{\partial t} \right]- \left( r_{D}\frac{\partial P_{D}}{\partial r_{D}} \right)_{r_{D}=1}$ | (12) |
| --- | --- |

The dimensionless wellbore storage coefficient is defined as (43):

| $C_{D}=\frac{v_{w} c_{w}}{2\pi h\emptyset c_{t}r_{w}^{2}}$ | (13) |
| --- | --- |

Since the wellhead pressure is constant, $\frac{\partial P_{tfD}}{\partial t_{D}}$ in (42) equals zero, with the definition of dimensionless production rate, (42) is simplified as follows:

| $q_{D}\left( t_{D} \right)=-C_{D}{(a}^{'}+S) {q_{D}}^{\mathbf{'}}+\left. q_{D} \right]_{C_{D=0}}$ | (14) |
| --- | --- |
